# Influencing factors of pregnancy-related anxiety in advanced maternal age primigravidas and the development and evaluation of personalized psychological nursing interventions

**DOI:** 10.3389/fpsyt.2025.1653736

**Published:** 2025-11-17

**Authors:** Yan Gao, Lei Yang, Jingwen Sun, Yiping Nan, Li Hou, Xiaomei Li

**Affiliations:** 1 School of Nursing, Health Science Centre, Xi’an Jiaotong University, Xi’an, China; 2 Department of Obstetrics, The Second Affiliated Hospital of Xi’an Medical University, Xi’an, China; 3 The School of Nursing and Rehabilitation, Xi’an Jiaotong University City College, Xi’an, China; 4 Nursing and Rehabilitation College, Xi’an Medical University, Xi’an, China

**Keywords:** advanced maternal age, primigravidas, pregnancy-related anxiety, psychological nursing, personalized intervention

## Abstract

**Background:**

Primigravidas of advanced maternal age (AMA) encounter distinct challenges throughout pregnancy and are particularly susceptible to heightened levels of anxiety.

**Objective:**

To identify the influencing factors of pregnancy-related anxiety in AMA primigravidas and to develop, implement, and evaluate a personalized psychological nursing intervention.

**Methods:**

Phase I involved a cross-sectional survey of 300 AMA primigravidas to identify anxiety-influencing factors. Phase II was a randomized controlled trial (RCT) with 160 AMA primigravidas assigned to either a personalized psychological nursing intervention group or a standard care control group.

**Results:**

Phase I identified key factors influencing anxiety, including perceived pregnancy risks, social support, and self-efficacy. In Phase II, the intervention group showed significantly lower anxiety levels (p < 0.001), improved self-efficacy (p < 0.001), and higher satisfaction with prenatal care (p < 0.01) compared to the control group.

**Conclusion:**

Personalized psychological nursing interventions based on identified influencing factors effectively reduce anxiety and improve psychological well-being in AMA primigravidas. Implementation of such interventions in prenatal care could significantly enhance outcomes for this vulnerable population.

## Introduction

The trend of delayed childbearing is increasingly prevalent in many developed and developing countries (1). This demographic shift is attributed to various factors, including increased educational and career opportunities for women, advancements in assisted reproductive technologies, and changing societal norms regarding family planning (2). Although pregnancies in women of advanced maternal age (AMA) are associated with numerous medical risks, the psychological aspects of pregnancy in this population, particularly among primigravidas (first-time mothers), have received less attention (3).

Pregnancy-related anxiety, defined as pregnancy-specific anxiety encompassing worries about fetal health, childbirth, and maternal role adaptation, has been shown to have unique implications for maternal and fetal well-being (4). In AMA primigravidas, these anxieties may be amplified due to the heightened awareness of age-related pregnancy risks and the pressure of motherhood at a later stage in life.

AMA primigravidas face a complex set of challenges, including increased medical risks of gestational diabetes, hypertensive disorders, and chromosomal abnormalities (5). The higher likelihood of requiring medical interventions or experiencing pregnancy complications can create a state of constant vigilance and worry. Additionally, psychosocial factors—such as employment or career concerns, financial stability, loneliness and perceived social pressure—may further compound the anxiety experienced by these women (6). The combination of medical and psychosocial factors creates a unique context for AMA primigravidas, highlighting the need to understand factors influencing pregnancy-related anxiety in this population. A systematic review identified pregnancy-specific, psychosocial, and personal determinants of prenatal anxiety (7). However, their applicability to AMA primigravidas remains uncertain. By addressing pregnancy-related anxiety effectively, there is potential to improve both maternal and fetal outcomes (8). Understanding these determinants provides the foundation for developing targeted psychological interventions specifically tailored to AMA primigravidas.

Despite growing recognition of prenatal anxiety’s impact on maternal health, most interventions have targeted general pregnant populations, with limited attention to the unique psychological needs of women of advanced maternal age (9, 10). Furthermore, the potential of personalized psychological nursing interventions—adapted to individual risk factors, psychosocial contexts, and personal preferences—remains underexplored in this specific group (11). We hypothesize that low social support, high prenatal distress, and low self-efficacy are key predictors of pregnancy-related anxiety among AMA primigravidas, and that a personalized psychological nursing intervention can significantly reduce anxiety levels.

Therefore, this study aims to explore the determinants of pregnancy-related anxiety among AMA primigravidas and to evaluate the effectiveness of personalized psychological nursing interventions tailored to their needs.

## Methods

### Study design

A mixed-methods approach was employed, consisting of two sequential phases. This design was chosen to provide a comprehensive understanding of the research problem and to enable the development and evaluation of an evidence-based intervention. In this study, the “mixed” component refers to the integration of quantitative data from the cross-sectional survey and randomized controlled trial (RCT) phases, complemented by limited qualitative feedback collected from participants in the intervention phase. Phase I utilized a cross-sectional survey design to identify and analyze factors influencing pregnancy-related anxiety in AMA primigravidas. This quantitative approach enabled the collection of standardized data from a large sample, facilitating the identification of statistically significant predictors of anxiety. Phase II involved a RCT to evaluate the effectiveness of a personalized psychological nursing intervention developed based on the findings from Phase I. The RCT design was selected for its robustness in establishing causal relationships between the intervention and outcomes, minimizing bias, and controlling for confounding factors.

### Setting and participants

The study was conducted at the Department of Obstetrics, Xi’an Jiaotong University First Affiliated Hospital, between January 2023 and December 2023. The hospital was chosen for its high volume of AMA primigravidas and diverse patient population, which enhanced the generalizability of the findings. Participants were recruited through antenatal clinics, where research assistants approached eligible women during routine prenatal visits and provided information about the study. Posters were also displayed in clinic waiting areas to facilitate voluntary participation.

Inclusion criteria for both phases were (1): primigravida status (2); maternal age ≥ 35 years at the expected delivery date, consistent with the definition of AMA; and (3) singleton pregnancy, to eliminate confounding from multiple gestations. Exclusion criteria included (1): diagnosed psychiatric disorders, to avoid confounding effects on anxiety measures (2); severe medical complications requiring intensive care; and (3) participation in other psychological intervention studies.

Ethical approval was obtained from the Ethics Committee of Xi’an Jiaotong University First Affiliated Hospital (Approval No. 2022-OB-PRA-015). Written informed consent was obtained from all participants prior to data collection.

### Randomization and blinding (only for phase II)

Participants who met the inclusion criteria were randomly assigned to either the intervention or control group in a 1:1 ratio. The randomization sequence was generated by an independent biostatistician using a computer-generated random number table. Allocation was concealed using sequentially numbered, opaque, sealed envelopes to prevent foreknowledge of group assignments. Participant enrollment was conducted by trained research nurses, while group assignment was performed by a separate investigator who was not involved in data collection or analysis.

Due to the nature of the psychological intervention, blinding of participants and intervention providers was not feasible. However, to minimize performance bias, all participants received standardized prenatal care and follow-up according to hospital guidelines, and intervention fidelity was ensured through staff training and protocol adherence monitoring.

Outcome assessors and data analysts were blinded to group assignments. Data were coded using anonymized participant identifiers, and a separate data entry team handled all questionnaire and outcome data to maintain blinding integrity throughout the analysis process.

### Data collection and outcomes measured

Data were collected using a structured questionnaire package that incorporated multiple validated psychometric instruments, each selected for its established reliability, validity, and relevance to the study objectives.

The instruments included:

#### Pregnancy-related anxiety questionnaire–revised

A 10-item scale measuring pregnancy-specific anxiety related to maternal concerns, fetal health, and delivery (12). This tool was selected for its specificity to pregnancy-related anxiety and demonstrated cross-cultural validity in Chinese populations.

Multidimensional Scale of Perceived Social Support (MSPSS):

A 12-item instrument assessing perceived support from family, friends, and significant others (13). As perceived social support is a well-established determinant of maternal mental health, this scale was included to capture the interpersonal dimension of well-being.

#### Prenatal distress questionnaire

A 12-item measure assessing pregnancy-specific distress, including concerns about physical symptoms, childbirth, and maternal role transition (14). The PDQ complements the PRAQ-R by encompassing broader emotional responses during pregnancy.

#### General self-efficacy scale

A 10-item scale evaluating an individual’s belief in their capacity to manage difficult situations (15). Self-efficacy is known to buffer stress and anxiety during pregnancy, justifying its inclusion.

#### Edinburgh postnatal depression scale

A 10-item screening instrument for depressive symptoms during pregnancy and the postpartum period (16). Given the high comorbidity between anxiety and depression, the EPDS provided a complementary measure of overall mental health.

Satisfaction with Prenatal Care Questionnaire (SPCQ):

Satisfaction with prenatal care was assessed using the Patient Satisfaction with Prenatal Care Questionnaire developed by Noonan et al. (17), which has been validated in maternal populations and shown to have strong internal consistency (Cronbach’s α = 0.89).

All instruments were administered in Chinese, following standardized translation and back-translation procedures consistent with WHO guidelines to ensure conceptual and linguistic equivalence. Where required (e.g., PRAQ-R, EPDS), formal permission and licensing for use were obtained from the instrument developers.

In addition to the standardized scales, a demographic and obstetric history form was used to collect information on age, education level, occupation, marital status, annual household income, pregnancy planning, and medical history. These variables were included based on prior evidence linking them to pregnancy-related anxiety.

To ensure confidentiality and minimize potential bias, data collection was conducted in private consultation rooms within the prenatal care clinics. Trained research assistants, blinded to participant allocation, provided clarification as needed without influencing responses.

### Interventions

The personalized psychological nursing intervention was developed based on the findings from Phase I and incorporated elements of cognitive-behavioral therapy (CBT), mindfulness, and psychoeducation. These evidence-based therapeutic components were selected for their established efficacy in managing anxiety and their adaptability to the unique needs of pregnant women.

The intervention was conducted over a six-week period, combining individual and group sessions to enhance engagement and skill application. It consisted of several components, each targeting key factors influencing pregnancy-related anxiety identified in Phase I:

1. Initial assessment: A comprehensive evaluation of individual anxiety-influencing factors and psychological needs was conducted using structured interviews and standardized tools.
2. Personalized care plan: Based on the assessment results, a tailored intervention plan was developed addressing identified risk factors and integrating patient preferences.
3. One-on-one counseling sessions: Six weekly 60-minute sessions were conducted by trained psychiatric nurses. The sessions applied CBT principles, including cognitive restructuring, stress management, and relaxation training.
4. Group support meetings: Bi-weekly 90-minute sessions focused on peer interaction and shared experiences to foster social connectedness.
5. Mindfulness and relaxation training: Participants received training in progressive muscle relaxation and guided imagery, supported by audio materials for home practice.
6. Educational materials: Pregnancy and parenting information tailored to AMA primigravidas was provided through printed and digital formats.
7. Partner involvement: Partners participated in selected sessions to enhance emotional support and shared coping. Partner inclusion has been shown to improve maternal psychological outcomes and relationship satisfaction during pregnancy (17).

Intervention fidelity was ensured through multiple measures. Session delivery followed a standardized manual, and nurses completed fidelity checklists after each session. Random session recordings were reviewed by a senior clinical psychologist for adherence. All psychiatric nurses held at least a Master’s degree in psychiatric or mental health nursing and had completed a 20-hour training program on CBT and mindfulness-based interventions before implementation.

A summary of intervention components and their corresponding objectives is provided in **Table 1**, which facilitates replicability and clarity for future applications.

**Table 1:** Summary of intervention components and corresponding objectives.

Component	Description	Objective
Initial Assessment	Structured evaluation of psychological needs, anxiety triggers, and coping resources through interviews and standardized tools.	To identify individualized risk factors and guide intervention tailoring.
Personalized Care Plan	Development of a customized plan based on assessment findings and participant preferences.	To ensure that intervention content is relevant and person-centered.
One-on-One CBT Counseling	Six weekly 60-minute sessions focused on cognitive restructuring, stress management, and relaxation training.	To reduce maladaptive thoughts and anxiety symptoms through cognitive and behavioral techniques.
Group Support Meetings	Bi-weekly 90-minute sessions promoting peer discussion and shared experiences.	To enhance social connectedness and normalize pregnancy-related concerns.
Mindfulness and Relaxation Training	Guided mindfulness, breathing, and muscle-relaxation exercises with take-home audio materials.	To improve emotional regulation and reduce physiological stress responses.
Psychoeducation	Evidence-based education on pregnancy, childbirth, and maternal adjustment tailored for AMA primigravidas.	To increase knowledge, self-efficacy, and sense of control.
Partner Involvement	Partners participated in selected sessions emphasizing communication and shared coping.	To strengthen emotional support and relationship satisfaction.
Monitoring and Fidelity Check	Use of standardized manuals, supervision, and session checklists.	To maintain consistency and intervention quality.

The control group received standard prenatal care, consisting of routine medical check-ups, general pregnancy education, and mental health referrals as needed.

### Sample size calculation

Sample size estimations were conducted separately for Phase I (cross-sectional survey) and Phase II (RCT). All calculations were performed using G*Power version 3.1.9.7.

For Phase I, the required sample size for proportion estimation was determined using:


$$\text{n}=\frac{Z^{2}\times p(1-p)}{d^{2}}$$

with Z = 1.96 (95% confidence level), p = 0.50 (estimated prevalence of pregnancy-related anxiety among AMA primigravidas), and d = 0.06. The minimum required sample size was 267, which was increased to 300 to account for non-responses.

For Phase II (RCT), the target was to detect a medium effect size (Cohen’s d = 0.5) between intervention and control groups, with power = 0.80 and α = 0.05 (two-tailed). Although the G*Power calculation indicates a minimum of 64 participants per group, to ensure adequate power and account conservatively for attrition and implementation variability, we set the final minimum at 80 participants per group (total N = 160).

### Statistics analysis

Descriptive statistics were used to summarize participant characteristics and questionnaire scores, providing an overview of the sample population and the distribution of key variables.

For Phase I, Pearson’s correlation coefficient was used to examine relationships between continuous variables, and multiple linear regression analysis identified factors independently associated with pregnancy-related anxiety (PRAQ-R scores as the dependent variable). Variables were entered into the model based on theoretical relevance and bivariate associations, using a stepwise approach to derive the most parsimonious model. Normality of data distributions was assessed using the Shapiro–Wilk test prior to applying parametric analyses.

For Phase II (RCT), an intention-to-treat approach was adopted to preserve randomization. Repeated measures ANOVA was used to evaluate within- and between-group changes in continuous outcomes (PRAQ-R, MSPSS, PDQ, GSE, and EPDS scores) over time, while χ² tests assessed categorical outcomes (e.g., mode of delivery, satisfaction with prenatal care). Independent t-tests were applied for continuous obstetric outcomes (e.g., gestational age, birth weight). Where assumptions of normality were violated, non-parametric alternatives (e.g., Mann–Whitney U) were employed.

Missing data were handled using multiple imputation to minimize bias due to attrition or incomplete responses. Additionally, effect sizes (Cohen’s d, partial η²) were reported alongside p-values to improve the interpretability of results.

Subgroup analyses were pre-specified in the study protocol to assess potential differences in intervention effectiveness by baseline anxiety level (high vs. low) and maternal age group (35–39 years vs. ≥40 years).

All statistical analyses were conducted using SPSS version 25.0 (IBM Corp., Armonk, NY, USA), with a two-tailed significance level set at p < 0.05. Assumptions for parametric tests were verified, and appropriate data transformations were applied when necessary.

## Results

The Results section is presented in two parts corresponding to Phase I (cross-sectional survey) and Phase II (RCT), ensuring a clear and logical flow of findings. All analyses were conducted according to the predefined statistical plan described in the Methods section.

### Phase I: cross-sectional survey

Of the 300 AMA primigravidas approached, 287 completed the survey (response rate: 95.7%), enhancing representativeness and minimizing non-response bias. The mean age of participants was 37.4 ± 2.3 years, consistent with the target AMA primigravidas. Demographic characteristics are presented in **Table 2**.

**Table 2:** Demographic characteristics of phase I participants (N = 287).

Characteristic	N (%)
Age (years)	
35-37	156 (54.4%)
38-40	98 (34.1%)
>40	33 (11.5%)
Education level	
High school or below	42 (14.6%)
College/university	189 (65.9%)
Postgraduate	56 (19.5%)
Employment status	
Employed	231 (80.5%)
Unemployed	56 (19.5%)
Marital status	
Married	268 (93.4%)
Single/Divorced	19 (6.6%)
Annual household income	
<$50,000	63 (22.0%)
$50,000-$100,000	152 (53.0%)
>$100,000	72 (25.1%)
Pregnancy planning	
Planned	201 (70.0%)
Unplanned	86 (30.0%)

The mean PRAQ-R score was 22.7 ± 6.8. Significant bivariate correlations were found between pregnancy-related anxiety and perceived social support (r = −0.42, p < 0.001), prenatal distress (r = 0.58, p < 0.001), self-efficacy (r = −0.39, p < 0.001), and depressive symptoms (r = 0.45, p < 0.001).

Multiple linear regression (**Table 3**) identified five independent predictors of pregnancy-related anxiety.

**Table 3:** Factors independently associated with pregnancy-related anxiety.

Factor	β	95% CI	P-value
Lower perceived social support	-0.31	-0.42 to -0.20	<0.001
Higher prenatal distress	0.35	0.24 to 0.46	<0.001
Lower self-efficacy	-0.22	-0.33 to -0.11	<0.001
Presence of depressive symptoms	0.18	0.07 to 0.29	0.001
Unplanned pregnancy	0.15	0.04 to 0.26	0.007
Lower education level	-0.12	-0.23 to -0.01	0.032

The model explained 47% of the variance in PRAQ-R scores (adjusted R² = 0.47), indicating strong explanatory power.

### Phase II: randomized controlled trial

A total of 160 AMA primigravidas were randomized equally to the intervention group (n = 80) and control group (n = 80). Baseline characteristics were comparable between groups (**Table 4**), confirming successful randomization.

**Table 4:** Baseline characteristics of RCT participants.

Characteristic	Intervention (n=80)	Control (n=80)	P-value
Age (years), mean ± SD	37.6 ± 2.4	37.2 ± 2.1	0.24
Education level, n (%)			
High school or below	12 (15.0%)	11 (13.8%)	0.82
College/university	52 (65.0%)	54 (67.5%)	
Postgraduate	16 (20.0%)	15 (18.8%)	
Employed, n (%)	65 (81.3%)	63 (78.8%)	0.69
Married, n (%)	75 (93.8%)	74 (92.5%)	0.75
Planned pregnancy, n (%)	57 (71.3%)	55 (68.8%)	0.73
PRAQ-R score, mean ± SD	22.9 ± 6.7	22.5 ± 6.9	0.70
MSPSS score, mean ± SD	60.4 ± 12.8	61.2 ± 13.1	0.68
PDQ score, mean ± SD	18.7 ± 5.9	18.3 ± 6.1	0.66
GSE score, mean ± SD	28.6 ± 5.2	29.1 ± 5.4	0.55
EPDS score, mean ± SD	8.3 ± 4.6	8.1 ± 4.4	0.77

PRAQ-R, Pregnancy-Related Anxiety Questionnaire-Revised; MSPSS, Multidimensional Scale of Perceived Social Support; PDQ, Prenatal Distress Questionnaire; GSE, General Self-Efficacy Scale; EPDS, Edinburgh Postnatal Depression Scale.

### Primary outcome

The intervention group exhibited a significantly greater reduction in pregnancy-related anxiety over time compared with controls (F = 28.6, p < 0.001). Between-group differences were significant at both post-intervention and follow-up (**Table 5**).

**Table 5:** Changes in PRAQ-R scores over time.

Time point	Intervention (n=80)	Control (n=80)	Mean difference (95% CI)	P-value
Baseline	22.9 ± 6.7	22.5 ± 6.9	0.4 (-1.8 to 2.6)	0.70
Post-intervention	17.3 ± 5.8	21.5 ± 6.5	-4.2 (-5.7 to -2.7)	<0.001
Follow-up	15.8 ± 5.3	20.9 ± 6.7	-5.1 (-6.8 to -3.4)	<0.001

Missing data were minimal (<3%) and addressed using multiple imputation. Results from imputed and complete-case analyses were consistent. Effect size for the between-group difference: Cohen’s d = 0.74 (large effect). Interaction effect: partial η² = 0.16, indicating a meaningful intervention impact.

### Secondary outcomes

Significant between-group differences were also observed for all secondary psychological measures (**Table 6**).

**Table 6:** Changes in secondary psychological outcomes.

Outcome	Group	Baseline	Post-intervention	Follow-up	Time × Group interaction
MSPSS	Intervention	60.4 ± 12.8	68.7 ± 11.2	71.3 ± 10.5	F = 18.9, p < 0.001
	Control	61.2 ± 13.1	62.5 ± 12.8	63.1 ± 12.6	
PDQ	Intervention	18.7 ± 5.9	14.2 ± 4.8	12.8 ± 4.3	F = 22.4, p < 0.001
	Control	18.3 ± 6.1	17.9 ± 5.8	17.5 ± 5.7	
GSE	Intervention	28.6 ± 5.2	32.4 ± 4.7	33.8 ± 4.5	F = 15.7, p < 0.001
	Control	29.1 ± 5.4	29.8 ± 5.2	30.2 ± 5.3	
EPDS	Intervention	8.3 ± 4.6	6.1 ± 3.9	5.4 ± 3.7	F = 12.3, p < 0.001
	Control	8.1 ± 4.4	7.8 ± 4.3	7.5 ± 4.2	

MSPSS, Multidimensional Scale of Perceived Social Support; PDQ, Prenatal Distress Questionnaire; GSE, General Self-Efficacy Scale; EPDS, Edinburgh Postnatal Depression Scale.

All between-group comparisons remained significant after multiple imputation. Effect sizes ranged from Cohen’s d = 0.45 to 0.70, representing medium-to-large magnitudes of improvement across psychological domains.

### Obstetric outcomes

No significant between-group differences were observed in obstetric outcomes (**Table 7**).

**Table 7:** Obstetric outcomes.

Outcome	Intervention (n=80)	Control (n=80)	P-value
Mode of delivery, n (%)			0.68
Vaginal	52 (65.0%)	49 (61.3%)	
Cesarean section	28 (35.0%)	31 (38.7%)	
Gestational age at birth (weeks), mean ± SD	38.7 ± 1.6	38.5 ± 1.8	0.45
Birth weight (g), mean ± SD	3285 ± 428	3247 ± 452	0.58

### Intervention adherence and acceptability

Of the 80 participants in the intervention group, 73 (91.3%) completed all six individual sessions, and 68 (85.0%) attended at least five of the six group meetings. Overall, 92.5% rated the intervention as “very helpful” or “extremely helpful”. Participants particularly valued the tailored psychological strategies, peer support, and partner involvement. These qualitative impressions were consistent with quantitative findings, underscoring the acceptability and perceived effectiveness of the intervention.

### Subgroup analyses

Subgroup analyses showed that the intervention was equally effective across baseline anxiety levels and maternal age subgroups (35–39 years vs. ≥40 years), suggesting broad applicability of the personalized psychological approach among AMA primigravidas.

Consistent results were obtained across all imputed datasets, confirming the robustness of findings. The subgroup effect sizes ranged from Cohen’s d = 0.42 (low-anxiety subgroup) to 0.71 (high-anxiety subgroup), both representing moderate-to-large effects, further validating the clinical significance of the intervention.

## Discussion

This study provides a comprehensive examination of the psychological challenges faced by AMA primigravidas, successfully identifying key determinants of pregnancy-related anxiety and evaluating the efficacy of a tailored psychological intervention. Our findings offer both theoretical insights and practical implications for maternal mental healthcare.

The investigation revealed several significant predictors of pregnancy-related anxiety in this population. Most notably, diminished perceived social support emerged as a crucial factor, corroborating existing literature that emphasizes the protective role of social networks in mitigating prenatal psychological distress (18). This finding underscores the importance of developing interventions that not only provide professional psychological support but also actively facilitate the strengthening of social connections and peer relationships among expectant mothers. Furthermore, the strong correlation observed between prenatal distress and anxiety highlights the complex interplay of psychological symptoms during pregnancy, suggesting that integrated approaches addressing stress, depression, and anxiety simultaneously may yield optimal outcomes.

Our results demonstrate a clear inverse relationship between self-efficacy and anxiety levels, providing empirical support for Bandura’s self-efficacy theory in the context of AMA primigravidas (19). This relationship indicates that enhancing women’s confidence in their ability to manage pregnancy-related challenges represents a promising avenue for anxiety reduction. Similarly, the association between unplanned pregnancy and elevated anxiety aligns with established research on the psychological impact of unintended pregnancies (20). These findings collectively emphasize the necessity of designing interventions that address both the emotional experiences and contextual circumstances of AMA primigravidas.

The randomized controlled trial conducted in Phase II provides compelling evidence for the effectiveness of the personalized psychological nursing intervention. The significant reduction in PRAQ-R scores observed in the intervention group, maintained throughout the third trimester, indicates the intervention’s durable benefits. We propose that these sustained effects may be mediated through several mechanisms: the development of enhanced coping strategies, improvement in self-regulatory capacities, and reinforcement of adaptive behaviors acquired during counseling sessions (21, 22). These mechanisms appear to foster psychological resilience, thereby enabling participants to manage stressors more effectively as they approach childbirth.

The intervention demonstrated benefits beyond primary outcome measures, with significant improvements observed across multiple secondary psychological domains. Reductions in prenatal distress and depressive symptoms, coupled with increased self-efficacy scores, affirm the multidimensional value of the approach. The high satisfaction ratings reported by participants further validate the intervention’s relevance and personal significance. These outcomes resonate with cross-cultural research indicating that culturally responsive, psychosocially oriented prenatal interventions can enhance maternal well-being across diverse settings (23, 24).

While the intervention did not significantly influence obstetric outcomes, the absence of adverse effects is noteworthy. This suggests that psychological improvements were achieved without compromising physical health parameters. Future longitudinal investigations should explore whether the reduction in prenatal anxiety translates to improved postpartum adjustment, enhanced maternal-infant bonding, and positive child developmental outcomes (25).

Several methodological strengths enhance the validity of our findings. The mixed-methods design enabled both comprehensive exploration of risk factors and rigorous evaluation of intervention efficacy. Additionally, the integration of empirically identified predictors into the intervention framework increased its precision and clinical applicability.

Nevertheless, certain limitations must be acknowledged. The single-center design and the predominantly educated, employed, and married sample may limit the generalizability of findings to more diverse populations. The reliance on self-report measures introduces the possibility of reporting and expectation biases. Although outcome assessors were blinded, participants’ awareness of their group allocation may have influenced subjective responses. The conclusion of follow-up during late pregnancy represents another limitation; future studies would benefit from including postpartum assessments to determine long-term intervention effects.

Beyond methodological considerations, our findings carry important implications for clinical practice and health policy. The integration of personalized psychological support into routine prenatal care—particularly for high-risk populations such as AMA primigravidas—holds significant potential for improving maternal mental health outcomes and patient satisfaction. With appropriate staff training and contextual adaptation, such interventions could be feasibly implemented within standard antenatal care programs, community health settings, and digital health platforms.

## Conclusions

In conclusion, this study demonstrates that a personalized psychological nursing intervention offers substantial benefits in reducing pregnancy-related anxiety and enhancing overall psychological well-being among AMA primigravidas. By targeting key risk factors—including low social support, high distress, and low self-efficacy—this approach provides an evidence-based, patient-centered model for prenatal psychological care.

Future initiatives should prioritize multi-center implementation, cultural adaptation, and integration into national maternal health policies to ensure broader accessibility and sustainable impact. Addressing the distinctive psychological needs of AMA primigravidas through structured, personalized interventions represents an essential advancement toward holistic and equitable maternal healthcare.

## Data Availability

The raw data supporting the conclusions of this article will be made available by the authors, without undue reservation.
